# The Pleistocene-Holocene aquatic molluscs as indicators of the past ecosystem changes in Transbaikalia (Eastern Siberia, Russia)

**DOI:** 10.1371/journal.pone.0235588

**Published:** 2020-09-18

**Authors:** Olga K. Klishko, Evgeniy V. Kovychev, Maxim V. Vinarski, Arthur E. Bogan, Georgi A. Yurgenson

**Affiliations:** 1 Institute of Natural Resources, Ecology and Cryology, Siberian Branch, Russian Academy of Sciences, Chita, Russia; 2 Transbaikalian State University, Chita, Russia; 3 Laboratory of Macroecology and Biogeography of Invertebrates, Saint-Petersburg State University, Saint-Petersburg, Russia; 4 Research Laboratory, North Carolina Museum of Natural Sciences, Raleigh, North Carolina, United States of America; University of California, UNITED STATES

## Abstract

Data on the historical change of the Transbaikalian malacofauna in the Neopleistocene and Holocene is presented. Aquatic mollusc shells from archaeological excavations of the ancient settlements dating from the Neolithic period to Medieval and also from a drill hole of the Neopleistocene alluvial deposits were collected. In total eight species of bivalve molluscs from the families Margaritiferidae, Unionidae, Lymnocardiidae, Glycymerididae [marine], and two gastropod species from families Viviparidae and Planorbidae were identified. These species were aged using radiocarbon dating. It was found that the species ranged in age from more than 50.000 to 2.080–1.210 years BP. Five species inhabited the Transbaikal region which are locally extirpated today. Their disjunctive ranges in the past included southern Europe and Western and Eastern Siberia to Transbaikalia and in the east to Far East and Primorye Territory of Russia. A remarkable finding is that of the bivalve genus *Monodacna*, which was found very far from its native range, the Ponto-Caspian region. The time of existence and extirpation of the thermophilic species of genera *Monodacna*, *Planorbis*, *Lanceolaria* and *Amuropaludina* corresponds to cycles of the warming and cooling in Pleistocene and Holocene according to regional climate chronological scales. These species can be used as palaeoclimate indicators. Change of the regional malacofaunal species composition is connected with the natural climatochron cycles in the Pleistocene and Holocene resulting in evidence for succession. In the course of this succession, these stenothermal species became extirpated on a regional level, decreasing their global ranges.

## Introduction

Freshwater molluscs, both gastropods (snails) and bivalves (clams, mussels), represent a very significant component of freshwater ecosystems throughout the world [1–3]. Since the mid-19^th^ century, shells of freshwater Mollusca have been recognized as an important source of data for knowledge of the past ecosystems, their taxonomic diversity, and physical and chemical characteristics (depth, temperature, salinity, and other indicators). The usefulness of palaeomalacological studies for palaeoecological reconstructions of the climate and landscape dynamics has been repeatedly stressed in the literature [4–9]. For instance, some taxa of bivalves (like the Unionidae, a diverse family with more than 840 recent species) are recognized as good indicators of the temperature regime and climate changes as well as the anthropogenic impact [10–15]. Historical shifts in distribution of particular species of freshwater Mollusca reflect past alterations in landscapes and connections of palaeobasins. The data on the past occurrences of mollusc species may be helpful for modern conservation efforts [16, 17].

Traditionally, most information on extinct molluscs is obtained in the course of geological and palaeontological investigations. However, there is another important source of primary data, namely, archaeological excavations, which often provide numerous remains of shells [18, 19]. However, to the best of our knowledge, the use of mollusc shells found during archaeological excavations for palaeoecological reconstructions is still rather limited (at least as compared to analogous studies carried out by palaeogeographers and palaeontologists).

The main goal of this study was to use the subfossil shells of aquatic Mollusca unearthed during long-term archaeological excavations and a borehole made in the Transbaikalia area (Eastern Siberia, Russia) for a better understanding of the process of the formation of the regional malacofauna in context of the past climate changes.

The Transbaikalian region (= Zabaikalsky Krai in Russian) belongs to the three large river basins of Northern Asia (Yenisei-Baikal, Lena, Amur) that determine the hydrographic uniqueness of this area. The three basins converge on the slopes of a relatively low (1236 m) watershed Pallas Mt. The highest peak of the area is located here. “BAM” Mt., is 3073 m a. s. l., the center of modern regional glaciation, with 39 small glaciers. The lowest elevation (292 m) is in the valley of the Amur River. During the Pleistocene glaciations, huge and deep glacier-dammed reservoirs (more than 900 m depth), with inter-basin connections in the territory of Transbaikalia, arose [20]. These connections may have served as a ‘corridor’ for aquatic mollusc penetrations from areas lying westward of Transbaikalia.

The region is characterized by a sharp and frequent spatial and temporal variability of climate, associated with the mountainous nature of the relief, the basin effect, vertical zonality, etc. [21–24]. In the regional climatic chronological scale of Transbaikalia, several climatic rhythms of cooling and warming at around 50–10 thousand years BP (Palaeolithic) and 10–1 thousand years BP (Mesolithic-Iron Age period) are recognized [24]. The alternation of periods of warming and cooling in the Pleistocene (300–10 thousand years BP) and Holocene (10–1 thousand years BP) in the north of Transbaikalia is clearly recorded by changing spore-pollen complexes of plant communities, which are the most sensitive to the dynamics of environmental factors [25–28].

The aims of our work are as follows. 1. To determine both taxonomic identity and the absolute age of subfossil remains of aquatic shells from Transbaikalia; 2. To reveal mollusc species able to serve as the climate change indicators; 3. To correlate the changes in the malacofauna species composition with the climatic rhythms in the Neopleistocene and Holocene of Transbaikalia.

## Material and methods

**Fig 1:**
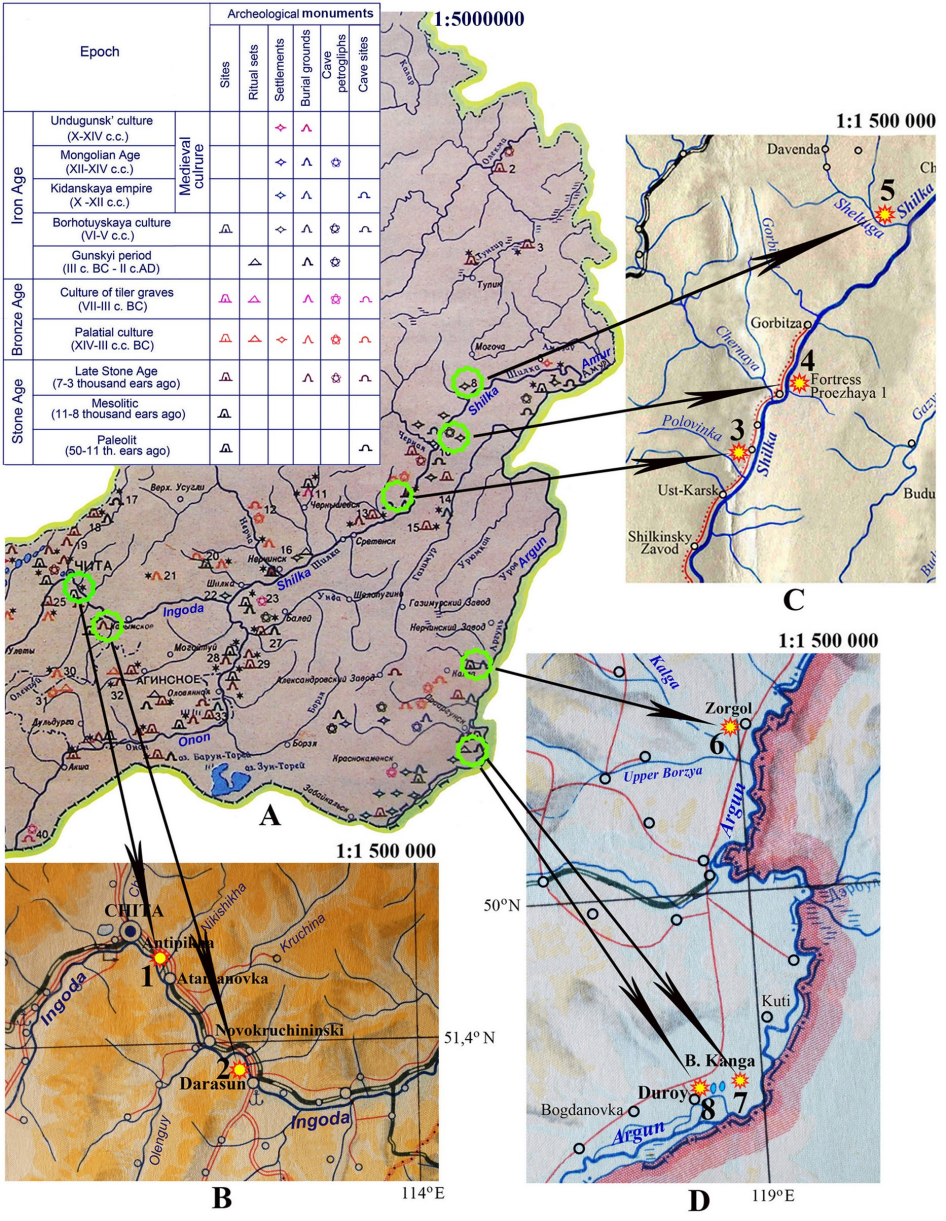
**A–**Archaeological map of Transbaikalia [32]; archaeological sites and monuments are designated by circles, sampling sites of shells–by numbers. **B1** –Chita district, Antipikha settlement, Ingoda River high-water bed, drill hole; **B2** –Karymsk district, Darasun settlement, ancient site. **C**–Sretensk district: **3 –**ancient Luzhanki settlement, **4 –**fortress Proezzhaya 1, **5 –**ancient settlement by Zheltuga River. **D**–Priargunsk district: **6** –Zorgol burial, **7 –**Bol’shaya Kanga ancient settlement 2; **8 –**Duroy 6 burial.

We examined the extensive conchological material collected in 1975–2019 during archaeological excavations in the territory of Transbaikalia [29–32]. These excavations have been carried out in archaeological sites, remains of settlements, and the burial grounds of prehistoric humans living in the Transbaikalia area (Fig 1A and Table 1). Some shells collected in 2019 from a drill-hole located in the Upper Amur Basin were also used. Most excavations were made under supervision of E.V. Kovychev and included the systematic study of archaeological objects and recovered artefacts. All investigated objects belonged to archaeological sites dated from the Stone Age to Medieval (11–3–1.2–0.2 thousand years BP). Excavations were carried out on stratigraphic blocks at depths between 0.8 and 6.0 m. Each occupation layer amounted to 10–15 cm thickness. All finds (bones of wild and domestic animals, ceramics, bitch bark, crafts made of bone and metal, fragments and unbroken mollusc shells) were sorted, classified, described, inventoried and dated.

**Table 1:** Geographical coordinates of the study sites in the Transbaikalia.

Sampling site	Coordinates
Chita district, Ingoda River palaeobed, Antipikha settlement, suburb of Chita city	51.994 N, 113.562 E
Karymsk district, right bank of the Ingoda River, near Darasun settlement	51.652 N, 113.967 E
Sretensk district:	
1. Right bank of the Polovinka River near Luzhanki settlement	52.758 N, 118.913 E
2. Proezzhaya 1 fortress, on the bank of the Shilka River	52.964 N, 119.102 E
3. Ancient settlement near Zheltuga River mouth	53.340 N, 119.622 E
Priargunsk district, Zorgol burial ground near Zorgol settlement	50.606 N,119.269 E
Priargunsk district, burial Duroy near Duroy lakes	50.011 N, 118.986 E
Priargunsk district, Bol’shaya Kanga ancient settlement	50.034 N, 119.099 E
Chita district, Kaidalovo village	51.624 N, 114.555 E
Kalar district, the Apsat coal deposit	57.054 N, 118.072 E

The absolute age of the excavated shells was determined by radiocarbon dating performed at the Institute of the Earth Sciences of the Saint-Petersburg State University, in the laboratory “Geomorphological and palaeontological investigations of the Polar regions and the World ocean”. The calibrated (calendar) age was determined by means of a calibrating software «OxCal 4.3».

The valves of mussel shells and their fragments were photographed from both the outside and the inside; the gastropod shells were photographed from different angles to reveal the diagnostically valuable traits. During taxonomic identification of the shell material, we compared it with phylogenetically closely related recent species from the Ingoda, Onon, Shilka, and Argun rivers located near the excavation sites. The size of the shells was restored by comparing their fragments with the hinges or muscle scars on valves from live collected specimens. In total, 257 shells and shell fragments from eight archaeological sites in the Upper Amur Basin were collected and investigated. The examined material is kept in the collection of the Transbaikal State University (TSU; Chita, Russia) and in the scientific collection of the Institute of Natural Resources, Ecology and Cryology of the Siberian Branch of the Russian Academy of Sciences (INREC SB RAS), Chita, Russia.

Abbreviation used: kya = thousand years ago; BP = Before Present

**Fig 2:**
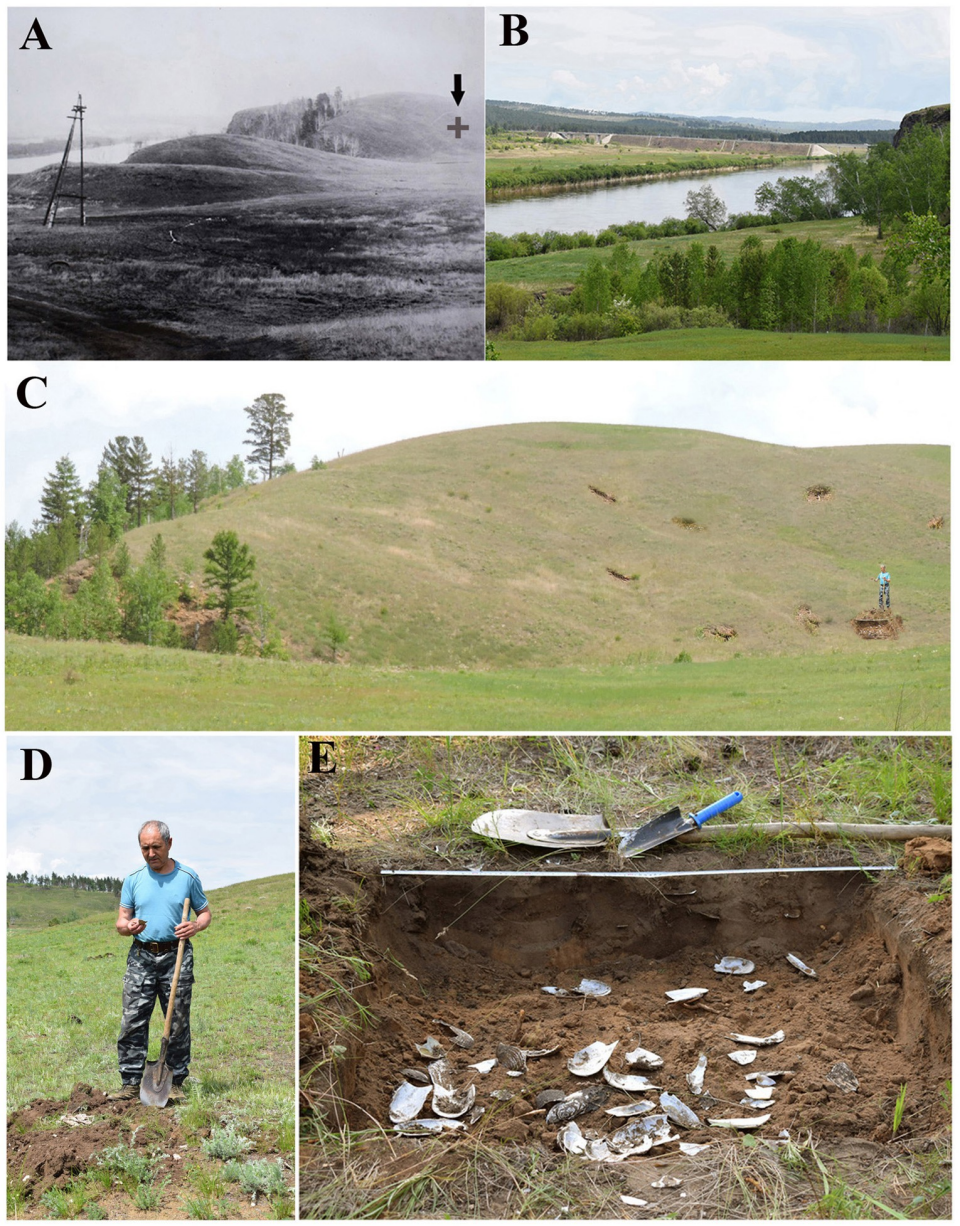
Excavations deposits on territory of the ancient site on a bank of the Ingoda River downstream of the Darasun settlement. А –location of this site identified in 1975 (marked by arrow **+**), В –appearance that location in 2019; С, D–trial excavation at the site in 2019; E–excavation deposits 3 with the layer of pearl mussel shells.

Ethic statement. The individual, whose image appears in Fig 2D of this manuscript, is one of the co-authors. He has given written informed consent to publish these case details.

## Results

### Systematic list of taxa

As a result of our examination of the shell collection, eight species of bivalve molluscs, including one marine species, and two species of gastropods belonging to gill-breathing and pulmonate snails, were identified. All these species are represented in the recent fauna though not all of them now live in the Transbaikalia waterbodies.

   Class **BIVALVIA**
  Family **Margaritiferidae** Henderson, 1929
Genus ***Margaritifera*** Schumacher, 1816
***Margaritifera dahurica*** (Middendorff, 1850)
  Family **Unionidae** Rafinesque, 1820
 Subfamily **Unioninae** Rafinesque, 1820
Genus ***Nodularia*** Conrad, 1853
***Nodularia douglasiae* (**Gray in Griffith & Pidgeon, 1833)
Genus ***Lanceolaria*** Conrad, 1853
***Lanceolaria grayii*** (Gray in Griffith & Pidgeon, 1833)
 Subfamily **Unioninae** Rafinesque, 1820
 Tribe **Anodontini** Rafinesque, 1820
Genus ***Cristaria*** Schumacher, 1817
***Cristaria plicata*** (Leach, 1814)
Genus ***Sinanodonta*** Modell, 1945
***Sinanodonta schrenkii*** (Lea, 1870)
  Family **Cardiidae Lamarck, 1809**
 Subfamily **Lymnocardiinae** Stoliczka, 1870
Genus ***Monodacna*** Eichwald, 1838
***Monodacna* cf. *polymorpha*** (Logvinenko et Starobogatov, 1967)
***Monodacna* cf. *colorata*** (Eichwald, 1829)
  Family **Glycymerididae** Dall, 1908
Genus ***Glycymeris*** (Sowerby III, 1889)
***Glycymeris* cf. *yessoensis*** (Sowerby III, 1889)–transported to area
   Class **Gastropoda**
  Family **Viviparidae** Gray, 1847
Genus ***Amuropaludina*** Moskvicheva, 1979
***Amuropaludina praerosa*** (Gerstfeldt, 1859)
  Family **Planorbidae** Rafinesque, 1815
Genus ***Planorbis*** Geoffroy, 1767
***Planorbis planorbis*** (Linnaeus, 1758)

### Historical records and modern range of species

#### Margaritifera dahurica

The earliest shell remains of Pearl Mussels (Margaritiferidae) in Transbaikalia, dated to the late Jurassic, were found in the coal seam of the Apsat deposit. These fossils were represented by shell imprints [33]. In the Neopleistocene, the existence of Pearl Mussels in the central Transbaikalia was affirmed by findings made in the Upper Amur Basin. The shells were found in the lower part of the alluvium section of the fourth terrace of the river Ingoda at the village Kaydalovo near the city of Chita [24]. The age of these finds was defined as Late-Middle-Pleistocene. The molluscs were identified as *Margaritifera dahurica*, as they looked similar to the modern representatives of this species. Further data on the fossil record of Pearl Mussels in the Upper Amur Basin was obtained from archaeological excavations of sites and ancient settlements of Transbaikalia, where *M*. *dahurica* shells were the most common and numerous. In particular, fossil shells of this mussel were discovered in an archaeological site situated on a high hill on the right descending bank of the Ingoda River, 4.5 km below Darasun settlement (Figs 1B and 2). This site was discovered in 1975 (Fig 2A), the excavation of the above mentioned plot was carried out in 2019. Fragments of shells were found randomly scattered over the settlement site (Fig 2C and 2D). In excavation No. 3, which is 1x1 m in size, in the subsoil layer, small and large fragments of shells were relatively concentrated (Fig 2E). Their lengths ranged from 3–5 to 9–13 cm. The total length of excavated shells, restored by comparing fragments with intact valves of the modern Pearl Mussels, could reach 16–20 cm (S1 Fig). The radiocarbon age of *Margaritifera dahurica* shells from this site was 1620±60 years BP, and the calibrated (calendar) age was 1510±70 years BP.

Pearl Mussel shells were also found in excavations of ancient sites and villages in the Shilka and Argun River basins (Fig 1C3, 1C4, 1C5 and 1D7), whose dates determined by archaeologists vary from the Neolithic to the Medieval Age (S2–S4 Figs). The radiocarbon age of their shells ranged from 2080±70 to 1690±70 years BP.

**Fig 3:**
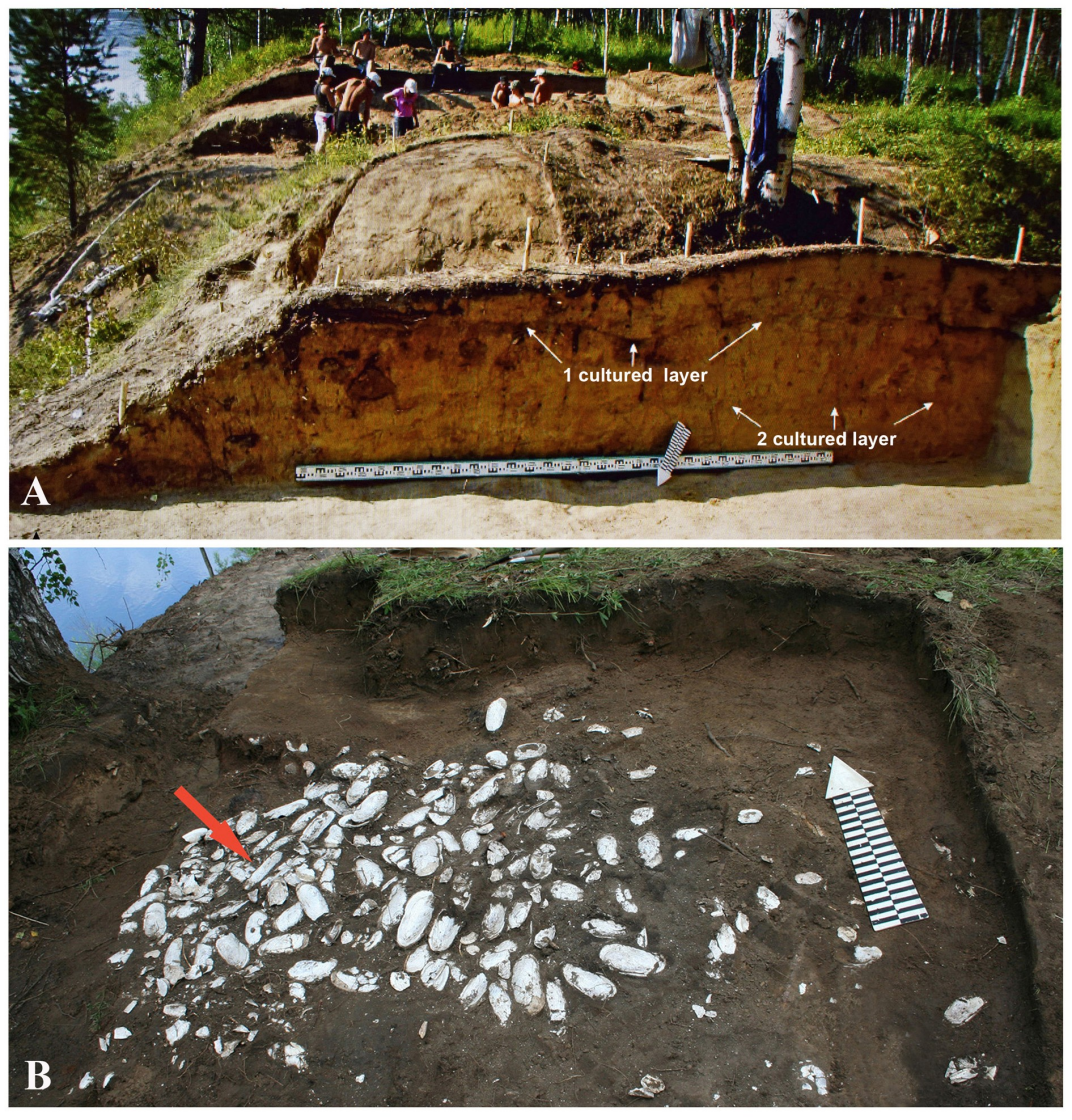
Archaeological excavations in the Proezzhaya 1 Fortress on the bank of the Shilka River. А –stratigraphic section of the excavation wall 47, in the background–excavation deposits of the dwelling 32, В –excavation of the outside slope bank of defensive fortification with clean layer of pearl mussel shells (*Lanceolaria* shells indicated by red arrow).

Shells of *Margaritifera dahurica* were most numerous in the excavations at Proezzhaya 1, a large ancient settlement located on the second floodplain terrace (6–9 m high) of the Shilka River, 4.2 km below Ust’-Chernaya village of the Sretensk district (Fig 1C4). Inside the 200 m long and 8 to 64 m wide area protected by defensive ramparts and ditches, the remains of 70 dwellings and 20 household pits were located. Dwellings were fixed in the form of quadrangular pits ranging in size from 4x4 m to 9x12 m. Bones of wild and domestic mammals, birds, fish, fragments of ceramics and aquatic mollusc shells were found in pits of the occupation layers (Fig 3A). In the dwellings themselves, shells were rare, usually they were found in household pits, outside the dwellings. For instance, in the excavation of the rampart of the defensive fortifications (Fig 3B), a layer of shells was found containing 147 fragments and whole shells of Pearl Mussels and three *Lanceolaria grayii* (shown by an arrow), but in pit No. 32 only three fragments of small margaritiferid shells were excavated (S2 Fig).

#### Lanceolaria grayii

**Fig 4:**
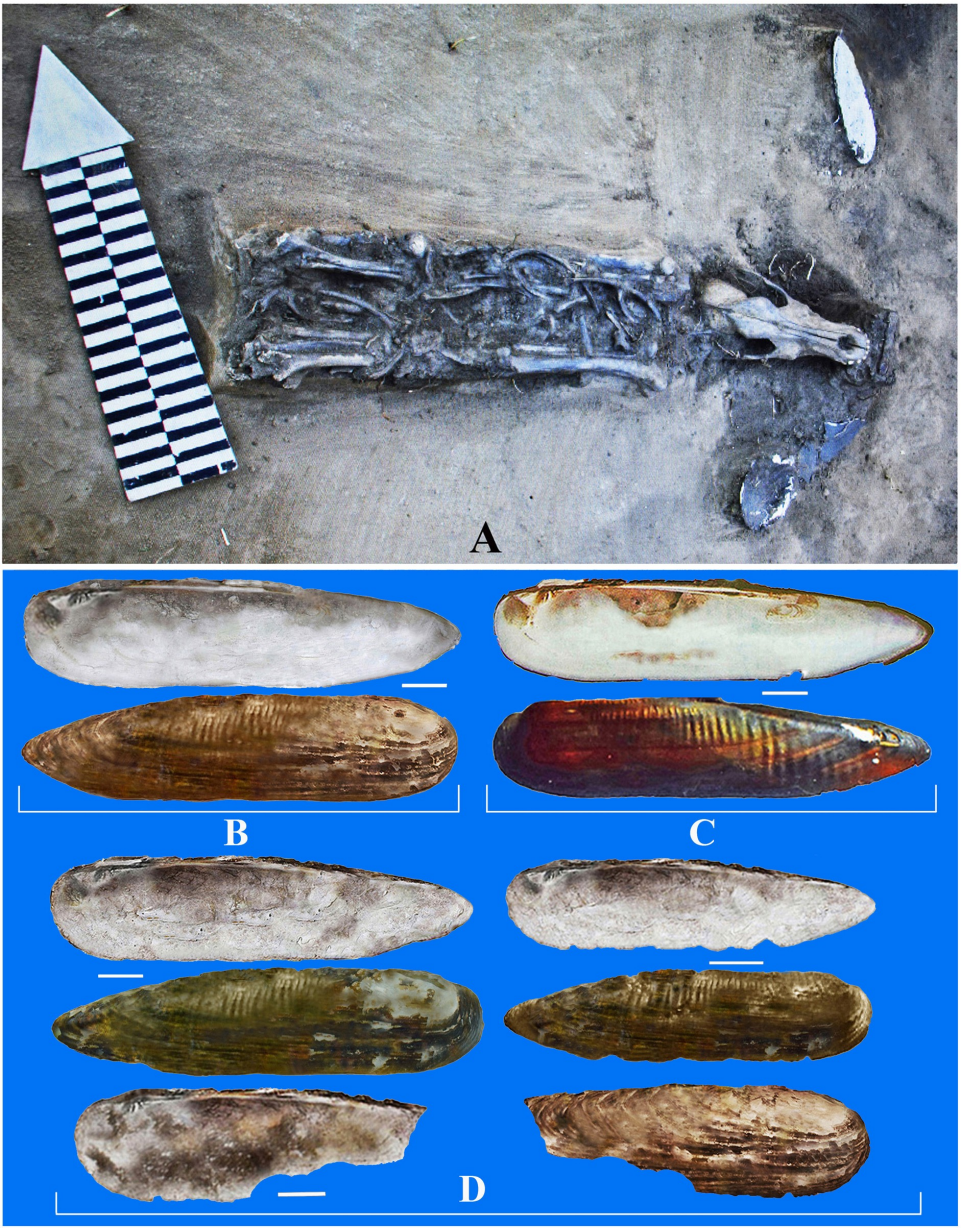
A ritual dog burial in the dugout 28 in the fortress Proezzhaya I. А –the dog skeleton, the left of his skull is a shell valve of *Lanceolaria grayii*, to the right of the dog skull–valve fragment of *Margaritifera dahurica*. В –*L*. *grayii* from the dog burial, С –recent *L*. *grayii* from Khanka Lake. D–shell valves of *L*. *grayii* from the bank of defensive fortifying excavation deposits. Scale bar 1 cm.

A rather large, narrowly elongated *Lanceolaria* shell was found in the ritual burial of a dog inside the dwelling 28 of the ancient settlement Proezzhaya 1. A juvenile dog skeleton was discovered during the excavation of the dugout floor. Shells of molluscs laid on both sides of its skull, and in front of it was an iron arrowhead (Fig 4A), which apparently represented ritual objects placed in the burial of a guard or hunting dog. It was assumed that the dead dogs were left in the dwellings intentionally to "guard" their inhabitants from intruders. Such dog burials were found in several dwellings of this settlement [31]. The radiocarbon age of the shell from the dog burial is 1550±80 years BP.

The valve of the *Lanceolaria* shell from the dog burial turned out to be identical in shape and hinge morphology with the modern *Lanceolaria grayii* from Lake Khanka (Fig 4B–4C), as well as shells from the fortification ramparts (Fig 4D). These archaeological finds from the Shilka River Basin were identified as *L*. *grayii*. According to molecular genetic studies, living mussels of the genus *Lanceolaria*, found in the Lake Khanka, Ussuri River Basin, and Lower Amur, belong to this species [34, 35].

#### Nodularia douglasiae

A fragment and an intact shell valve of *Nodularia*, 4.7 and 5.9 cm long, were found during the excavations of the Neolithic settlement 2 of Bol’shaya Kanga, in the Argun River Basin (Fig 1D7). The radiocarbon age of shells from this excavation was 2080±70 years BP.

**Fig 5:**
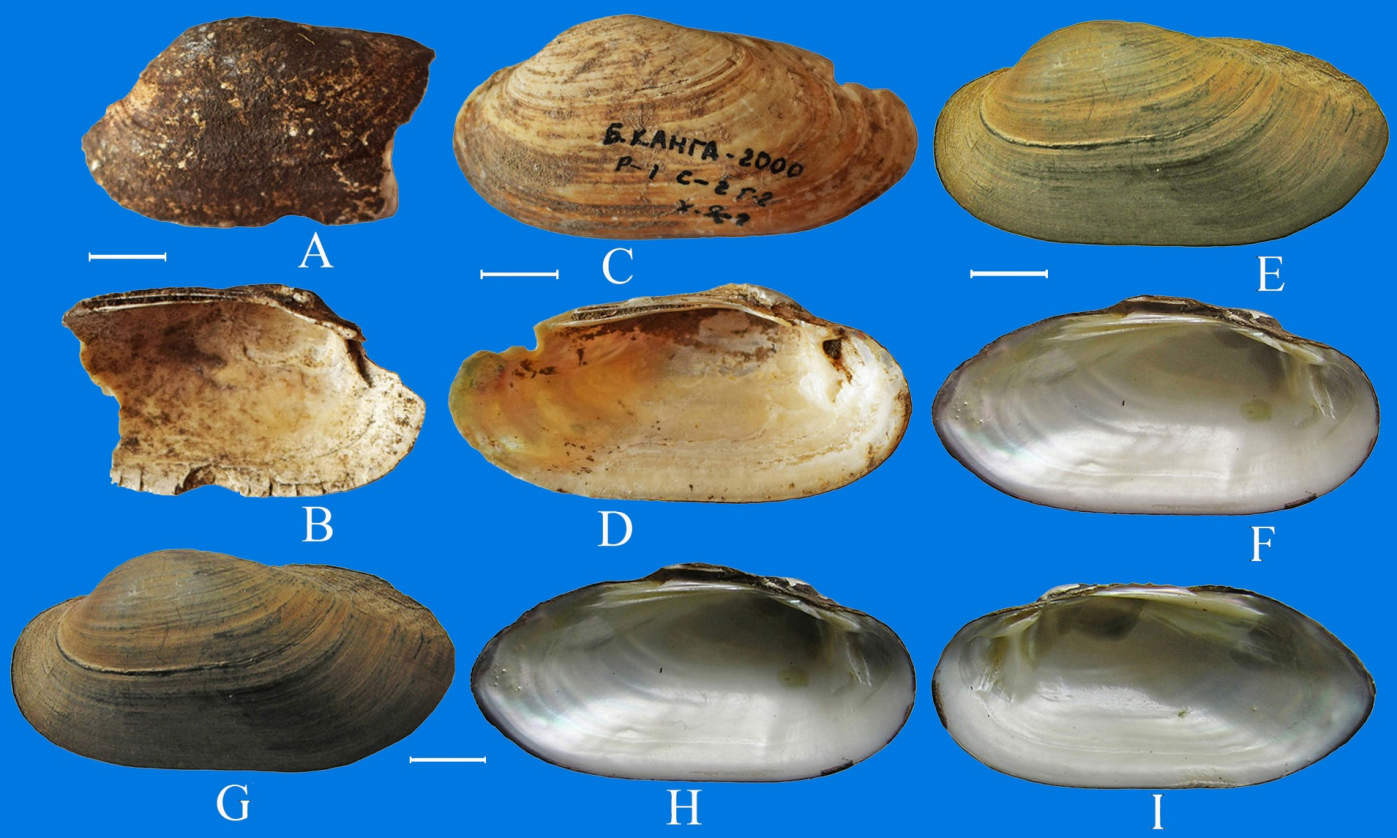
Subossil and recent shells of *Nodularia douglasiae*. A-D–shells (view on the outside and on the inside) from excavation of the Bol’shaya Kanga ancient settlement (late Stone Age), E, F–recent *N*. *douglasiae* from the Argun River and G-I–from the Ingoda River. Scale bar 1 cm.

Based on the complete morphological likeness of the shape, pseudocardinal and lateral teeth of shells from archaeological finds (Fig 5A–5D) and modern *Nodularia douglasiae* from the Argun and Ingoda rivers (Fig 5E–5I), these archaeological remains were identified as *Nodularia douglasiae*. This mussel species is widespread throughout the Amur River Basin, from the Magadan Region in the north to Sakhalin Island and the Primorye Territory of Russia in the south; it occurs also in Southeast Asia [13, 35].

#### Cristaria plicata

Scattered fragments of the anodontine *Cristaria* shells were collected from excavations in the Neolithic village of Bol’shaya Kanga, Argun River Basin (Fig 1D7), and an archaeological site on the right bank of the Polovinka River near Luzhanki settlement (Fig 1C3). Both archaeological finds date from the Neolithic (7–3 thousand years BP). The radiocarbon age of the shells from these excavations was 2080 ± 70 and 1690 ± 70 years BP.

**Fig 6:**
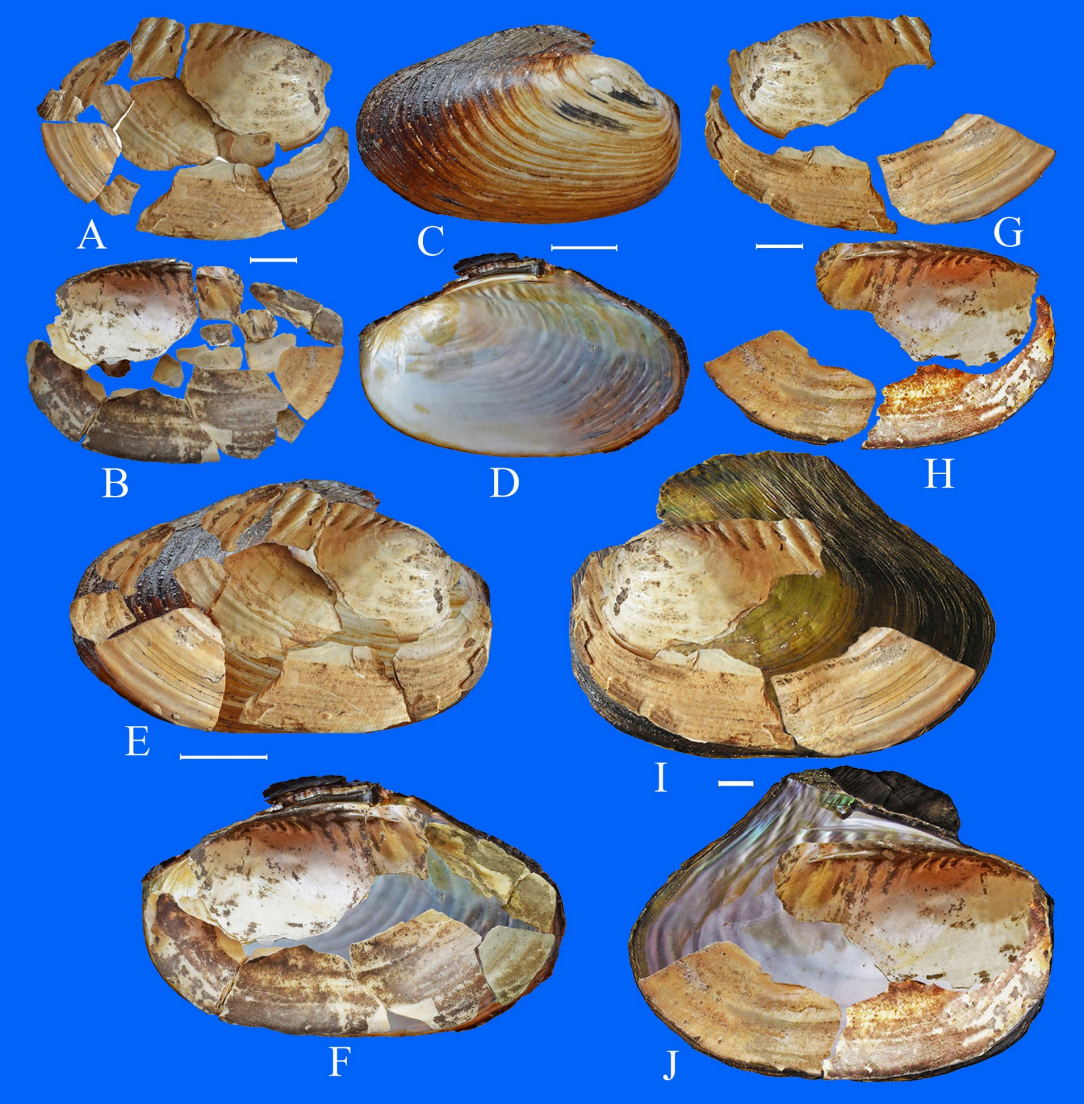
Archaeological specimens and recent shells of *Cristaria*. А, В –fragments of shell valves (views of the outside and inside) from archaeological excavation in the Bol’shaya Kanga, G-H–shell fragments of *Cristaria* from excavation in Luzhanki ancient settlement; С, D–recent *C*. *plicata* (view of the outside and inside of the shell) from the Shilka River; E-F–shell fragments from excavation in the Bol’shaya Kanga, superposed with a recent shell of *C*. *plicata* from the Shilka River; I-J–shell fragments of *Cristaria*, superposed with recent shell of *C*. *plicata* from the Onon River Basin. Scale bar 2 cm.

Reconstruction of shell valves was performed by comparing the excavated shell fragments (Fig 6A–6B) with valves of recent *Cristaria plicata* from the Shilka River (Fig 6E–6F) and the Onon River Basin (Fig 6G–6H). The lengths of anodontine shells could be 21–29 cm in accordance with the sizes of valve fragments with lateral tooth and the size of the same portion of the shell of modern *C*. *plicata* (Fig 6E–6F and 6I–6J). Now this species is widespread in the tributaries of the Amur River, in the Ussuri River Basin and Lake Khanka (Russia). It occurs also in Lake Buir-Nur (Mongolia) and countries of Southeast Asia [11].

#### Sinanodonta schrenkii

**Fig 7:**
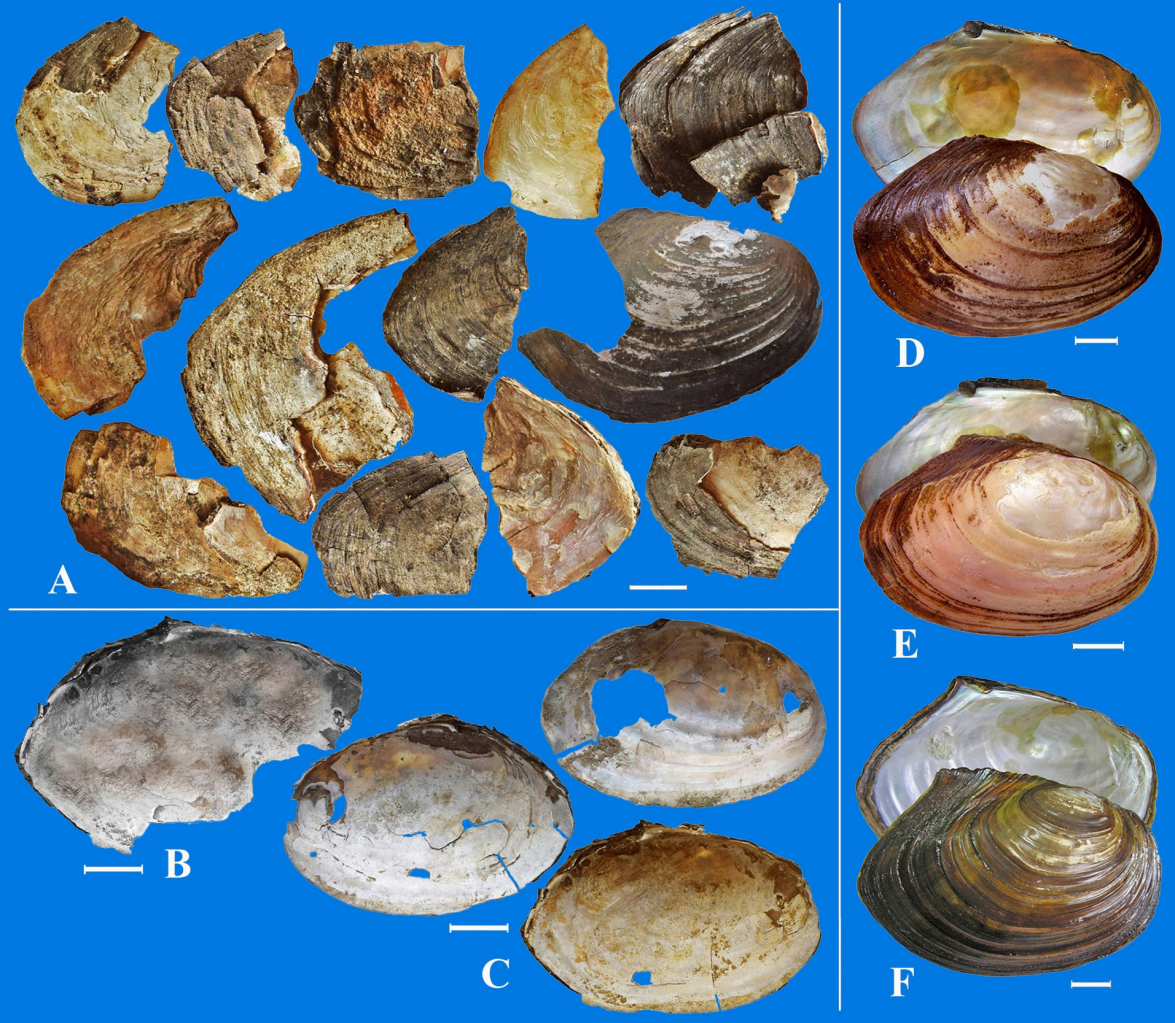
Archaeological specimens and recent shells of *Sinanodonta*. A–shell fragments of *Sinanodonta* from excavation in the Luzhanki settlement, В –from the Zheltuga settlement, C –from the Bol’shaya Kanga settlement. Recent shells of *S*. *schrenkii*: D–from the Shilka River, E–from the Nercha River and F–from the Ingoda River. Scale bar 2 cm.

Numerous intact shells and small shell fragments of the anodontine bivalve of the genus *Sinanodonta* together with abundant shells of Pearl Mussels were found in excavations of a site situated opposite Luzhanki settlement (Fig 1C**3**), an ancient settlement near Zheltuga River outlet (Fig 1C**5**) and the Bol’shaya Kanga settlement (Fig 1D**7**). The radiocarbon age of these shells was determined as 1690±70, 1770±90 and 2080±70 years BP, respectively. Size of fragments and intact shell valves varied from 8 cm to 15 cm (Fig 7A–7C).

Since the excavated shells and shell fragments were morphologically similar to recent shells of *Sinanodonta schrenkii* from the Amur River Basin, we identified them as belonging to this species. According to molecular genetics analyses, the five nominal species of the genus *Sinanodonta* inhabit the Amur River Basin and the Primorye Territory of Russia are conspecific and represent the intraspecific forms of a single valid species, *S*. *schrenkii* [34, 35], which is widespread in the Amur River Basin and the Primorye Territory of Russia.

#### Monodacna cf. polymorpha

**Fig 8:**
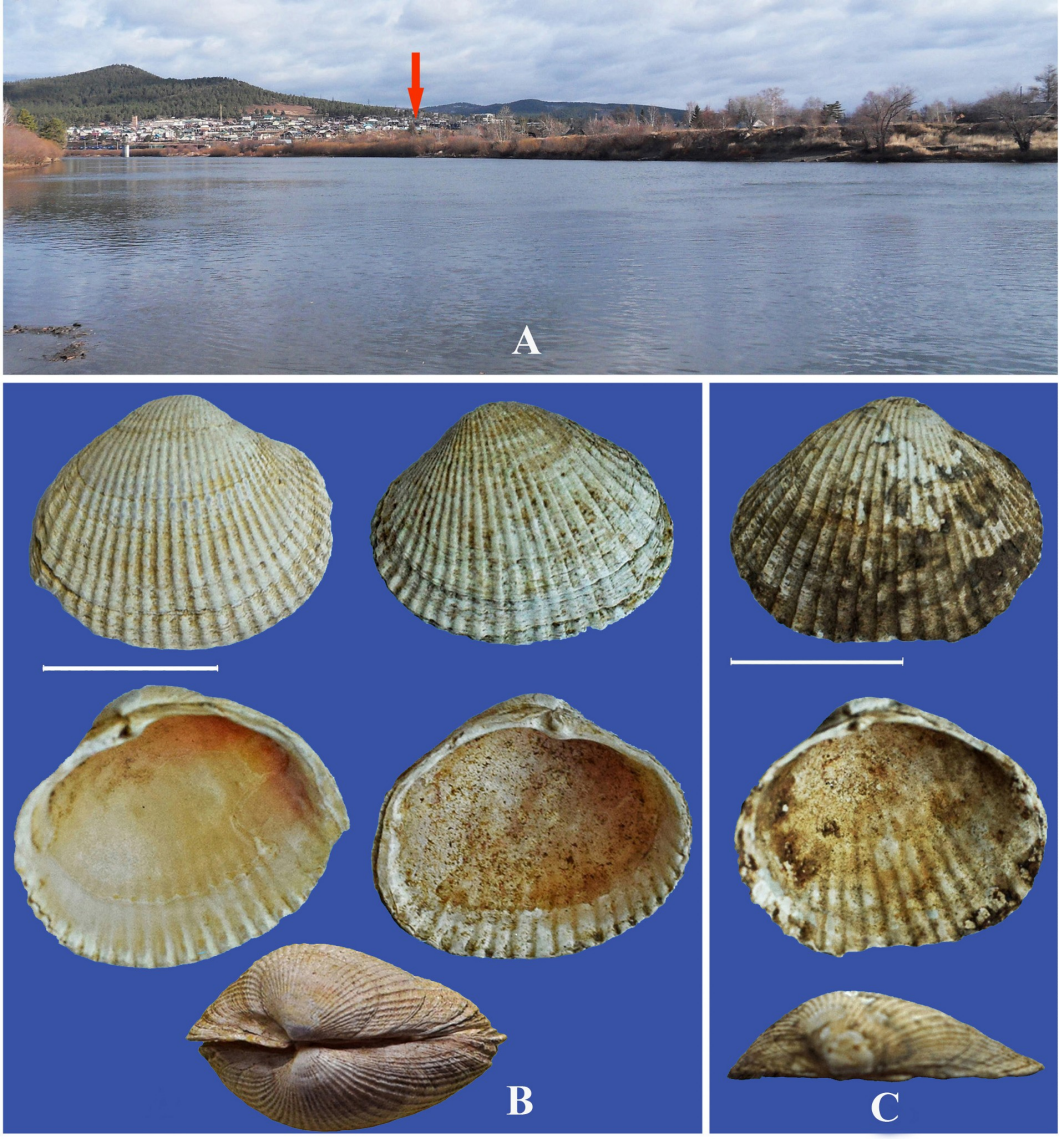
Locations of *Monodacna* species. A–Ingoda River near Antipikha settlement, suburb of Chita city. Fossil shells (Outside, inside and dorsal views) from drill hole in the Ingoda River flood plain: B–*Monodacna* cf. *polymorpha*, C–*Monodacna* cf. *colorata*. Scale bar 1 cm.

*Monodacna* cf. *colorata*. Shells of two cardiid bivalves of the genus *Monodacna* were found in the floodplain of the Ingoda River, on the territory of Antipikha settlement, a suburb of Chita City (Fig 1B**1**). The width of the floodplain in this place reaches 1.5 km; the site where the shells were found is situated 500 m from the current riverbed (Fig 8A). One shell was found in 2009 during excavation of a well; three valves of *M*. cf. *polymorpha* and one of *M*. cf. *colorata* (valve length 16–18 mm) were collected in 2019 at the same place from a soil drillhole (diameter 16 cm, depth 6–8 m). The shells, morphologically similar to recent *M*. *polymorpha* and *M*. *colorata*, are rather thick, convex, ovate-triangular, with narrow smoothed umbo, large triangular cardinal tooth, and shallow muscular scars and mantle sinus. Shell surface of *M*. cf. *polymorpha* is covered by narrow and frequent smoothed ribs, the width of which exceeds the width of the interrib intervals (Fig 8B). The ribs on shells of *M*. cf. *colorata* are wider, with acute not smoothed ribs (Fig 8C). The absolute age of the bivalve shells cannot be determined by radiocarbon dating but it must surely exceed 50 kya (for details see *Planorbis planorbis* section and Discussion). The genus *Monodacna* is a part of the Ponto-Caspian malacofauna; modern day representatives of this genus live only in the basins of the Black, Caspian, and Aral Seas [36, 37].

*Glycymeris* cf. *yessoensis*. *Glycymeris yessoensis* belongs to the marine bivalve family Glycymerididae. A shell of *Glycymeris* cf. *yessoensis* was found in 1991 in the burial ground on a terrace-like plateau near Duroyskiye lakes in the Argun River Basin (Fig 1D**8**). In total, 12 archaeological sites were discovered in this area. Among them–the Palaeolithic settlement of Duroy, belonging to the 1^st^ half of the Sartan glaciation (25–13 thousand years BP), where bones of a woolly rhinoceros, bison, mammoth, reindeer and stone tools were found. In the burial ground dated to the late Neolithic–Early Bronze Age, under the oval stone mounds of a diameter of 8–9 m, in grave pits 2.5–3 m deep, tribes living in the 3^rd^ century BC–2^nd^ century AD buried their dead [38]. The burial ground funerary equipment included fragments of clay vessels, stone products, animal bones, and shells of river molluscs. A shell of *Glycymeris* cf. *yessoensis* 42 mm long, with a hole in the umbonal area made for the sake of using it as a pendant, was discovered. The radiocarbon age of the shell was 5540 ± 160 years BP, the calibrated age was 4820 ± 130 years BP.

**Fig 9:**
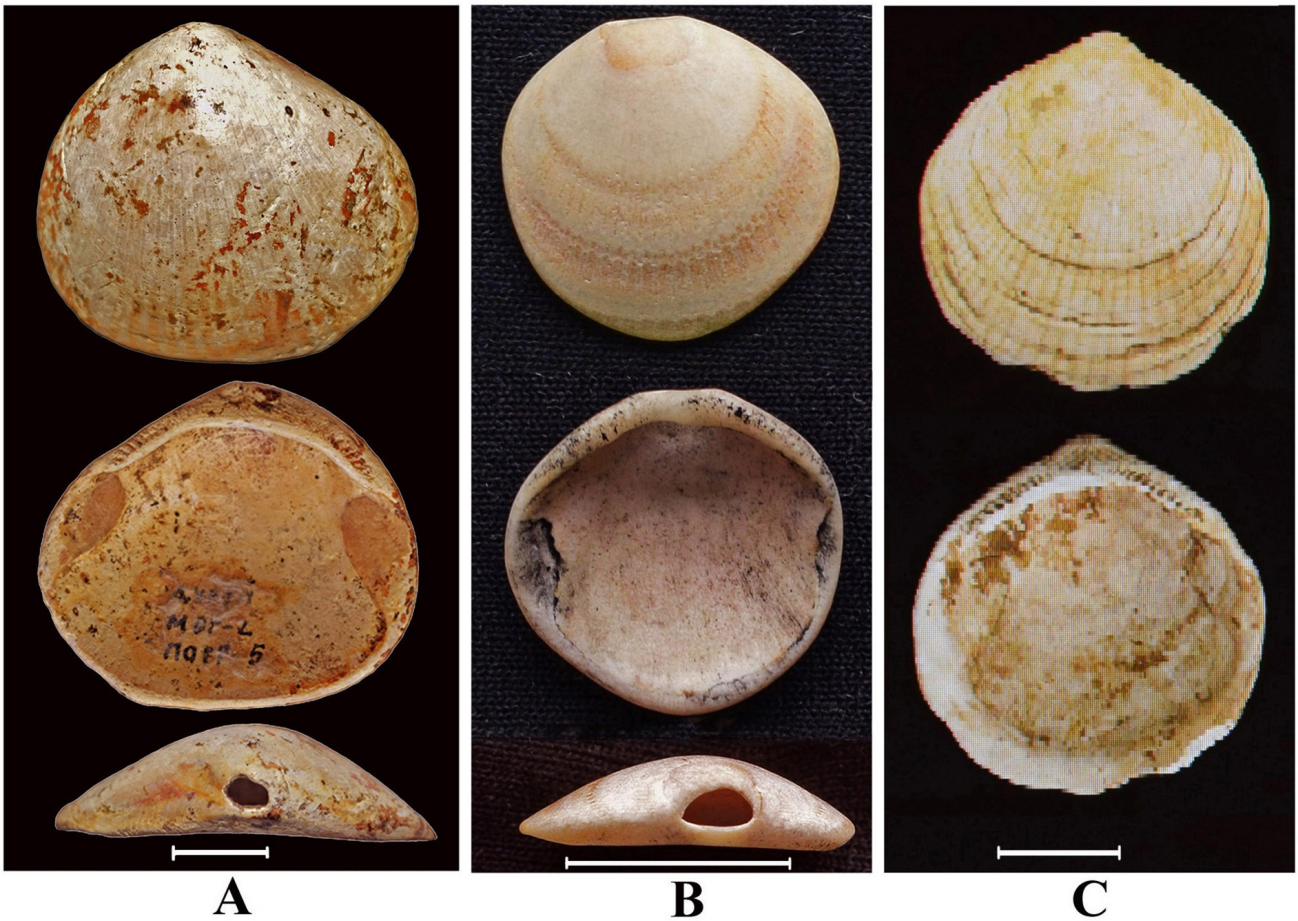
Archaeological specimens and recent shells of *Glycymeris*. Outside, inside and dorsal views: **A** –*G*. cf. *yessoensis* from the Duroy burial ground in Transbaikalye, **B** –recent *G*. *yessoensis* from the Amursky Bay (Sea of Japan/East Sea), the hole in the umbonal area of valve (dorsal view); **C** –*G*. *yessoensis* from excavation of the medieval settlement Nikolaeskoe I in the Primorye, Russian Far East [39]. Scale bar 1 cm.

According to the overall shell morphology, the shape of muscle scars and the mantle sinus, the shells from the Duroy burial ground are similar to recent *G*. *yessoensis* from the Amursky Bay (Sea of Japan/East Sea) (Fig 9A and 9B). The shell of *G*. *yessoensis* from the excavation of the medieval settlement Nikolaevskoe I in Primorye [39] also resembles the shell from the Duroy burial site, but differs by the symmetrical structure of the valves and the location of the mantle sinus slightly above the ventral edge (Fig 9C). In samples from the Duroy burial ground and the Amursky Bay, the posterior margin of the shell is slightly longer than the anterior one; the mantle sinus is located very close to the ventral margin of the shell. *Glycymeris yessoensis* is a common species of the South Kurile Islands, in shallow waters, found in the Amursky Bay (Sea of Japan/East Sea) [40, 41].

#### Amuropaludina praerosa

**Fig 10:**
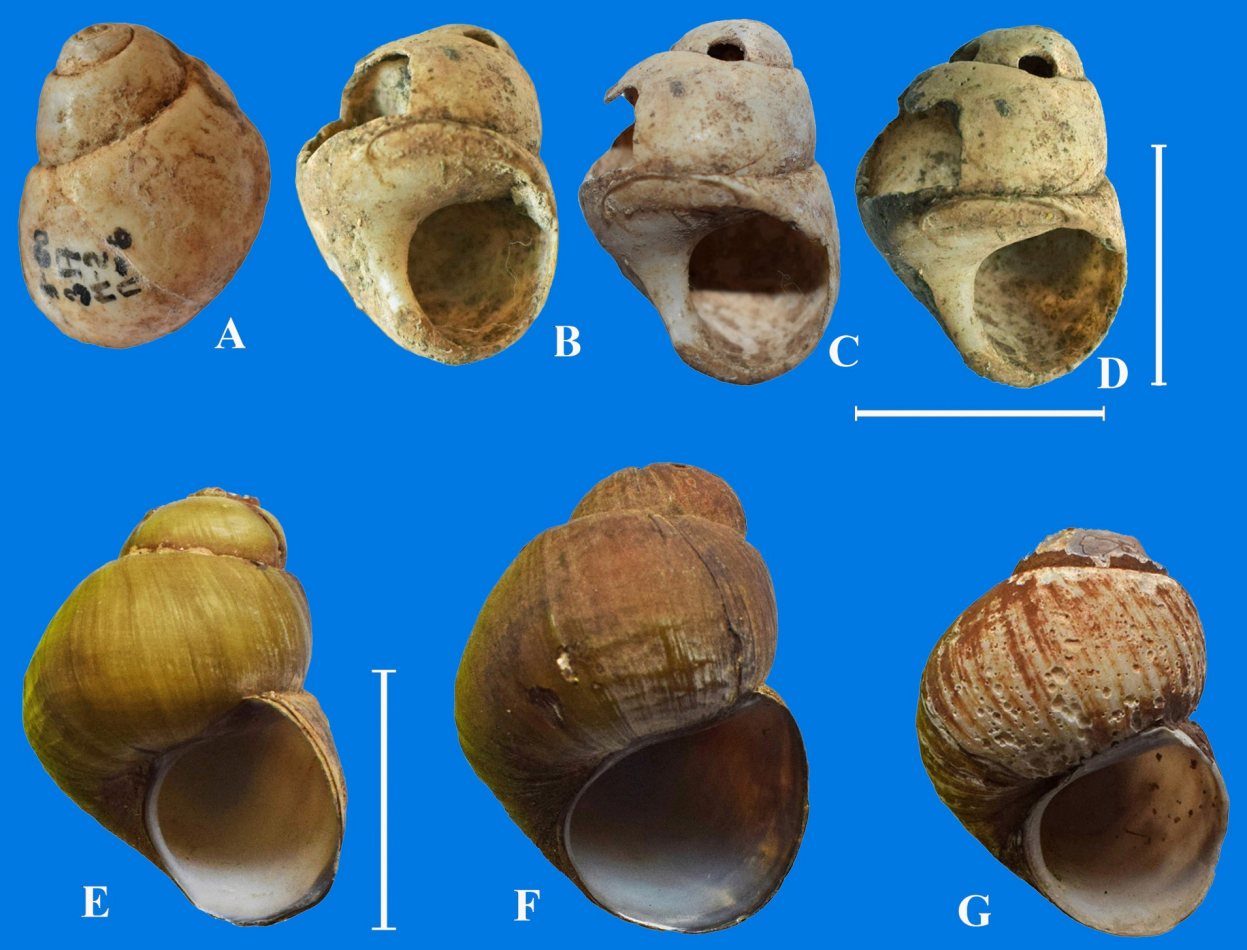
Shells of *Amuropaludina praerosa*. A-D–view of a subfossil shell *A*. *praerosa* in different perspective from the Zorgol burial ground, E-G–recent shells of *A*. *praerosa* from the Middle Amur River. Scale bar 2 cm.

A gastropod shell, morphologically similar to shells of the recent *A*. *praerosa* from the Amur River Basin, was discovered in the burial ground of Zorgol in the burial 6/4, dated I century. BC–I century AD. The absolute age of this shell by radiocarbon dating is 2160 ± 90 years BP. The Zorgol burials in the Priargunsk district (Fig 1D6) are represented by oval stone depositions, with grave pits of a rectangular shape 2–3 m deep. The burial items included clay and birch bark dishes, bone and iron arrowheads and spears, horn covers of bows, and various adornments [42]. Bones of sacrificed animals and shells of aquatic molluscs were found in the fill of burial pits and inside burials. The thick-walled high-turreted shell found in the burial, 31 mm high and 22 mm wide, had two holes in the upper whorl, which appear to be made for the purpose of using it as a pendant (Fig 10A–10D).

Living *A*. *praerosa* of similar size were collected in 2006 from the Middle Amur River near Poyarkovo settlement (Fig 10E–10G). Currently, the distribution of this species is limited to the middle and lower parts of the Amur Basin [36]. It does not occur in the Transbaikalia area [43].

#### Planorbis planorbis

**Fig 11:**
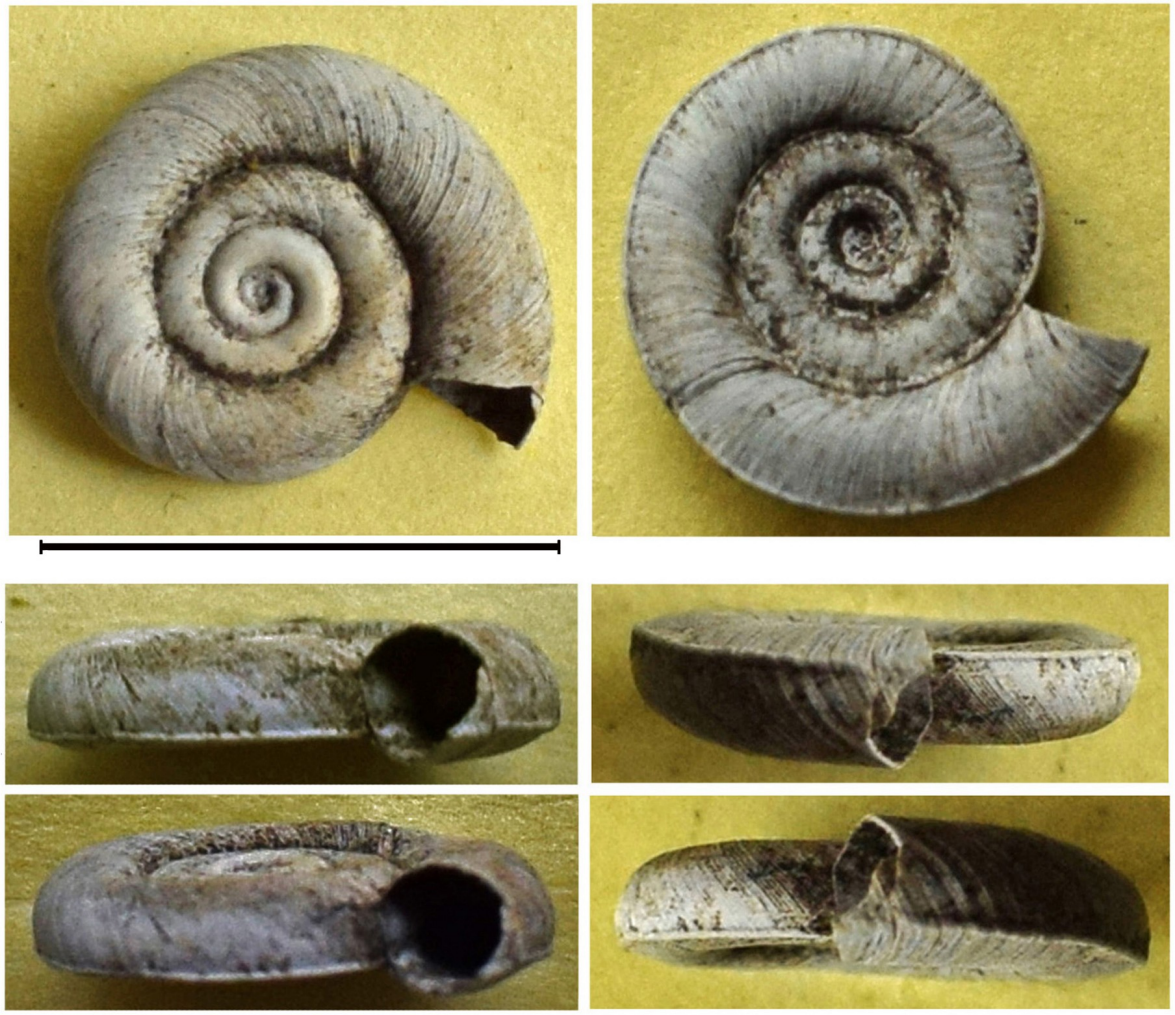
Fossil specimen of *Planorbis*. Shell from the drill hole with Pleistocene deposits in the flood plain of the Ingoda River.

The shell of the gastropod *Planorbis planorbis* from the alluvial deposits of the Ingoda River was extracted from a borehole located in vicinities of Chita City (Fig 1A1). This find was accompanied by shells of the cardiid genus *Monodacna* (see above). This shell bears all characteristics of Recent representatives of this species: it is rather small, discoidal, covered by fine axial striations. Shell whorls are slightly convex and increase relatively quickly and evenly, the last whorl is approximately twice the width of the penultimate one (Fig 11). A pronounced keel is visible along the basal surface of the last whorl, the aperture is evenly rounded, without distinct angulations. Shell dimensions (in mm) are: width 9.8, height 2.2.

We were unable to determine the absolute age of shells from this locality (both *Monodacna* and *Planorbis*) because of limited resolution of the radiocarbon dating method. The upper age of an object may be older than 50 kya because of a relatively short half-life of the ^14^C isotope.

**Fig 12:**
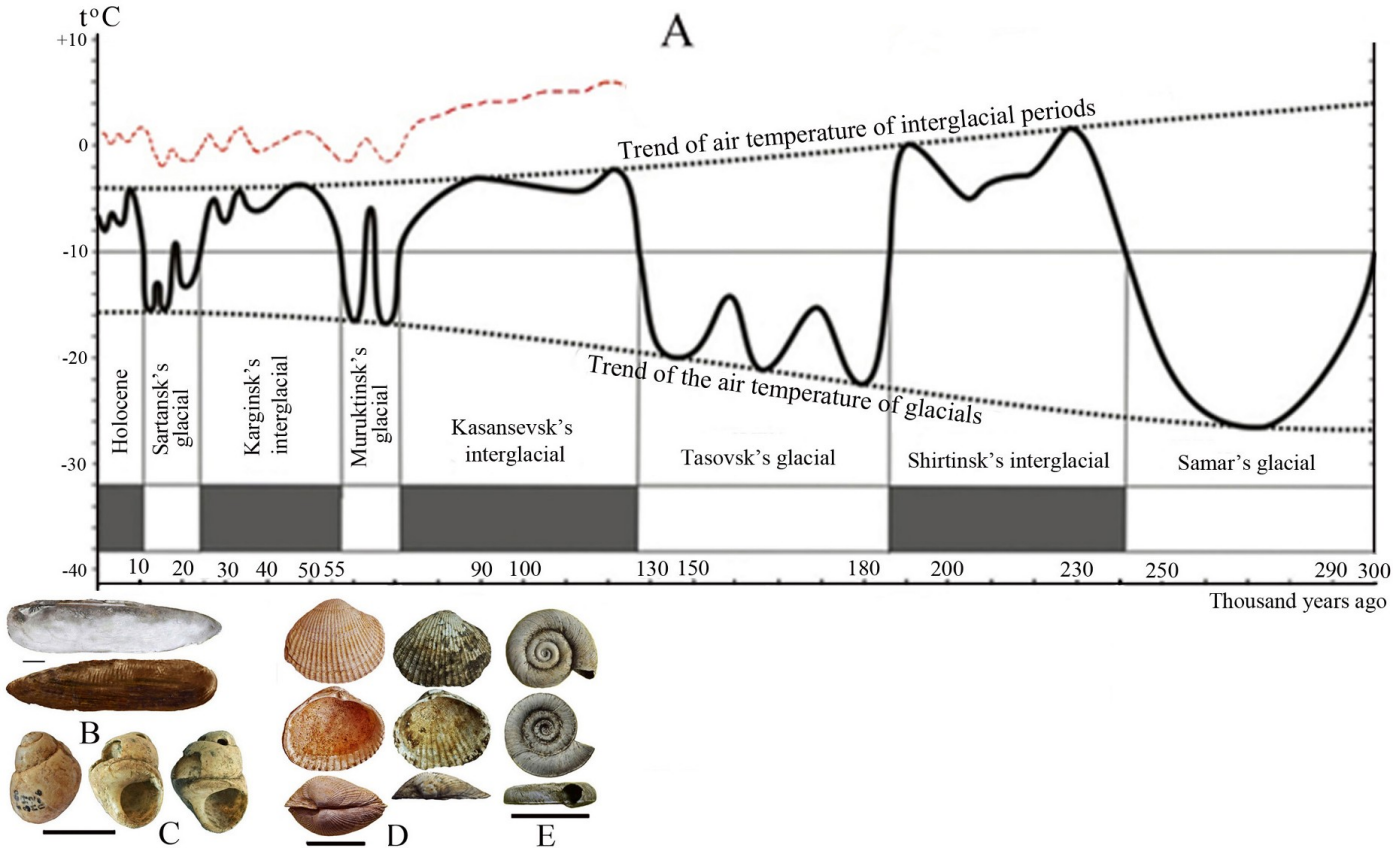
A–Change of the average annual temperature of ground air in epochs of Neopleistocene and Holocene according to palynological analyses of the core in Charskaya depression in the northern Transbaikalye [28, 57]. Red dashed line–a probable temperature trend in the Upper Amur Basin in the south-eastern Transbaikalye. Possible time of existence of thermophilic species in the Upper Amur Basin: **B** –*Lanceolaria grayii* и **C**–*Amuropaludina praerosa*, **D**–*Monodacna* cf. *polymorpha* and *Monodacna* cf. *colorata*, **E**–*Planorbis*. Scale bar for mussels = 1 cm.

Most probably, the existence of both *Monodacna* and *Planorbis* in Transbaikalia can be assigned to the Kazantsevsky interglacial of the Neopleistocene, in the time interval from 130–70 kya (see Fig 12. Discussion).

*Planorbis planorbis* shells are known from the late glacial lacustrine sediments of Chany Lake, southern part of the West Siberian Plain [44]. This species also was found in the Pleistocene sediments of the Kirenga River Basin in the Eastern Siberia [45]. These sediments were represented by gravel pebbles with a lens of green sands in the Kirenga River palaeobed. According to Popova [45], the age of *P*. *planorbis* in the Baikal area should not be older than the Lower–the early Middle Pleistocene, as indicated by spore-pollen complexes from this location, represented by pollen of spruce (*Picea excelsa*), cedar (*Pinus sibirica*), pine (*Pinus silvestris*), fir (*Abies* sp.), larch (*Larix* sp.), linden (*Tilia* sp.), oak (*Quercus* sp.), elm (*Elmas* sp.) and hemlock (*Tsuga* sp.), according to Kultchitski et al. [46]. The recent range of *P*. *planorbis* covers much of Europe and Western Siberia eastward to Altai Mountains [36, 37, 47]. It is absent from the Baikal Lake Basin and adjacent areas [48], though old records of this species (under the name *Planorbis marginatus*) from the shallow bays of the Baikal Lake are known [49, 50]. During faunistic surveys of freshwater Mollusca of the south part of Eastern Siberia, including Lake Baikal, this species has not been found (M. Vinarski, pers. observ.). It is reasonable to assume that *P*. *planorbis* is extirpated now from the study area.

### The Neopleistocene-Holocene molluscs as indicators of climate change

To assess the value of the studied mollusc species as possible indicators of the palaeoclimatic conditions, we compared the absolute age of identified species of gastropods and bivalves and the climatic phases (climatochrones) of the regional geochronological scale. In the Holocene epoch (Mesolithic-Iron Age period), between 10.0–2.0 kya, four climatic periods were distinguished from the Boreal to the Subatlantic (Table 2). In the Neopleistocene (Palaeolithic-Early Mesolithic), the Sartansky cryochron (25.0–10.8 kya) included 4 cool and 3 warm stages. The early Palaeolithic Age of Transbaikalia coincided with the Karginsky thermochron dated 50–25 kya. According to archaeological data and radiocarbon dating, the period of occurrence of the studied mollusc species in the Upper Amur River Basin does not coincide with the stages of the regional climatochronological scale.

**Table 2:** General geochronological scale for Late Pleistocene and Holocene with regional climate chronological scale [24], subdivisions of archaeological periodization [51] and radiocarbon dating of the mollusc shells (by our data).

Epoch (phase)	Regional climate chronological scale			Archeological periodization	Radiocarbon dating of shells (^14^C), in years BP
	Climatic chronology	Stage/period	Geochronolo-gical borders (thousand years)	Archaeological and geochronological borders	Species (age) № LU
		Medieval	0.12–0.10	X-XII century AD	*M*, L* (1210±60, 1550±80) LU-9665, LU-9661
Holocene (early late)	Holocene thermochron	Subatlantic	2.8–2.0	Late Bronze- early Iron (VII-III c. BC, VI-X c. AD)	*M*, *C*, *S* (1770±90–1690±70) LU-9662-9664-9666
		Subboreal	4.0–2.8	Early Bronze (XIV-III c. BC)	
		Atlantic (optimum)	8.0–4.0	Late Stone Age (6.5–3.8)	*M*, *N*, *S*, Am* (2160±90–2080±70) LU-9667-9669
				Late Mesolithic (8.0–6.5)	
		Boreal	10.0–8.0	Middle Mesolithic (10.3–8.0)	*M* (4820±130)
Pleistocene (late)	Sartanski cryochron	Cryostage	10.8	Early Mesolithic (10.8–10.3)	-
		Thermostage	12.0		
		Cryostage	12.2	Late Palaeolithic (18.0–10.8)	-
		Thermostage	12.7		
		Cryostage	15.0	Middle Palaeolithic (25.0–18.0)	-
		Thermostage	16.0		
		Cryostage	25.0		
	Karginski thermochron	Thermostage	30–25	Initial Palaeolithic (35.0–25.0)	*-*
		Cryostage	33–30		Mon*, P* (>50000) LU-9670

*M*–*M*. *dahurica*, L*–*Lanceolaria*, *N*–*Nodularia*, *C*–*Cristaria*, *S*–*Sinanodonta*, Am–Amuropaludina^*^, *Mon*–Monodacna^*^, *P*–Planorbis^*^, *–locally extirpated species.

Obviously the palaeogeographic situation in the southeast of Transbaikalia (50–52°N, 114–120°E) with a low-mountainous relief was significantly different from that in its northern high-mountainous regions (56.5°N), where powerful glaciation occurred in the Neopleistocene [52, 53]. According to data of Dr. F. Enikeev (Institute of Natural Resources, Ecology and Cryology SB RAS, personal communication), there was no a continental glacier in the southeast region, of the territory of the Upper Amur River Basin, but there were huge ice-dammed reservoirs interconnected with the vast glacial basins of Eurasia. The climate changes in this area were less drastic than in the north of the region.

According to palaeontological data, the Pearl Mussels (Margaritiferidae) in the Pleistocene of central Transbaikalia existed during cold climatic phases [24]. The archaeological findings show that *Margaritifera dahurica* lived in the southeast of Transbaikalia, in the Upper Amur River Basin from the Sartansky cryochron to the Holocene thermochron. This characterizes *M*. *dahurica* as an eurythermic species. *Lanceolaria grayii* and *Amuropaludina praerosa*, according to radiocarbon dating, occurred in the Upper Amur River Basin during the Holocene thermochron, and disappeared here during the late Holocene cooling, when the average annual air temperatures dropped below the optimum (+1.0–1.8°C), where these species currently live in the Far East of Russia (46–52°N, 130–140°E). Both *Lanceolaria grayii* and *Amuropaludina praerosa* can be characterized as stenothermal molluscs. The *Monodacna* species are now confined to the Ponto-Caspian area and occur in the basins of the Black, Caspian, and Aral seas. Their current range assumes the relative thermophily of these cardiids. This is a unique find for the region, which shows that the range of the genus *Monodacna* in the Neopleistocene was, probably, much wider. Possibly, the isolated part of the ranges was located in the Upper Amur basin in the Transbaikalia region. This question requires a further investigation. *Planorbis planorbis*, with its wide distribution, is, probably, the most eurythermic species of all discussed in this paper. Its collection from northern latitudes of Europe and Western Siberia are known [54–56] and, thus, the performance of this snail as a climate change indicator is very low. We can assume that *Monodacna* and *Planorbis* lived in the Upper Amur River Basin, probably during the Karginsky and Kazantsevsky interglacials of the late Pleistocene and were extirpated there during the Murukta or Sartan glaciation. The fact of the existence of these species in Eastern Siberia in the Neopleistocene suggests their wider ranges with peripheral and isolated (?) populations in the Transbaikalia area. The rest of species identified by us from archaeological samples (*Nodularia douglasiae*, *Cristaria plicata*, *Sinanodonta schrenkii*) survive in this region up to modern time, we consider them eurythermal and eurybiontic.

The amplitude of climate change in Transbaikalia during the Neopleistocene and Holocene is evidenced by palynological studies that record cycles of glaciation and deglaciation over a long period of time (up to 300 kya) [25, 52]. According to palynological analyses of 1.2 km of core in the Chara depression in the north of Transbaikalia (56.5°N, 116°E) [25, 52], the average annual air temperature in the Neopleistocene varied from +1 to –4°C, in the Holocene from –4 to –8°C, and during glacial stages from –16 to –22°C. (Fig 12). The age of the palynological complexes, confirmed by isotopic dating, shows a rather detailed picture of the dynamics of temperature indicators in the north of Transbaikalia, and the change in the species composition and the timespan of the fossil malacofauna in the Upper Amur River Basin reflect the dynamics of climate change in the southeast of Transbaikalia.

The significant heterogeneity of landscape and climatic conditions and the altitudinal position of the regions in Transbaikalia caused the mosaic formation of malacofauna both in the past and in the present, with a limited distribution of stenoecous species in the Upper Amur River Basin, which experienced less sharp climate fluctuations than the north of the region. When reconstructing the living conditions of recovered molluscs, the actualistic method of analogy based on knowledge of ecology and life habits of recent closely related species can be recommended [57].

The species *Lanceolaria grayii* and *Amuropaludina praerosa*, which are now extirpated in Transbaikalia, inhabited the Upper Amur River Basin 1550±80–2180±90 years BP, under milder climatic conditions similar to the modern climate of the Russian Far East. Their range covered the territory from the Upper to the Lower Amur River Basin, Ussuri River Basin and Lake Khanka. *Monodacna* and *Planorbis* lived here in the Neopleistocene, probably from 130 to 70 kya, in a warmed shallow lake or in a floodplain waterbody of the Ingoda River, in a neutral or slightly alkaline environment, on soft silty-sandy soils, summer water temperature could be around 22–24°C (by analogy with today’s environment of waterbodies of the Ingoda River floodplain). Although most representatives of the genus *Monodacna* are brackish water species, the modern invasion of *Monodacna colorata* in the lower and middle reaches of the Volga [36] shows that this species is not stenohaline and can exist under strictly freshwater conditions.

These molluscs, as indicator species, provide some information on climate change in the southeast of Transbaikalia during the Holocene and the Neopleistocene.

## Discussion

Shells and shell fragments of the Pearl Mussel *Margaritifera dahurica* were the most numerous among the mollusc remains found during archaeological excavations in Transbaikalia. The massive aggregations of its shells in archaeological sites, ancient households and hillforts (Figs 2 and S2–S4) suggest that the prehistoric humans collected these mussels mainly as a food item. The shells were also used for handicrafts and jewelry made of mother of pearl (buttons, arrowheads and spearheads, spinners, disk-shaped beads and bracelets) (S5 Fig).

The species of *Lanceolaria*, *Nodularia*, *Cristaria* and *Sinanodonta* are much rarer (Figs 4B–4D, 5A–5D, 6A–6B and 7A–7C), though the ancient people could also use them for food and as a material for crafts and decorations. The finding of a pendant made of *Amuropaludina praerosa* shell (Fig 10A–10D) in the Zorgol burial ground indicates that this species inhabited the Argun River Basin nearly 2.1–1.6 kya in milder climatic conditions as compared to the current ones.

An interesting find was a shell of the marine bivalve species, *Glycymeris* cf. *yessoensis*, in the Duroy burial ground (Fig 9A). It also was used as a pendant, which, perhaps, served as an offering and then a ritual object during the burial. The shell of *Glycymeris* cf. *yessoensis* from the burial place turned out to be quite similar to shells of *Glycymeris yessoensis* from the excavation of the medieval Nikolayevskoye 1 settlement in Primorye (Fig 9C). Possibly, the ancient tribes of the Amur and Primorye areas had transport and trade connections, which resulted in the shell of *Glycymeris* (of non-local origin) being found in Transbaikalia. This serves as an important complement to archaeological information about the lifestyle and culture of the ancient people of Transbaikalia.

The finding of shells of *Monodacna* and *Planorbis* (Figs 8B–8C and 11) from a borehole located in the Ingoda River floodplain is unique for Transbaikalia since these molluscs do not occur here anymore. Their current ranges are situated much to the west, in the southern part of Europe and Central Asia (*Monodacna* and *Planorbis*) and Western Siberia (*Planorbis*). It shows that their ranges were recently much wider. Possibly, the isolated parts of their ranges were located in Transbaikalia. Though the disappearance of the cardiid clams from the Baikal area may be easily explained by climate cooling and the disruption of the former interbasin connections, the regional extirpation of *Planorbis* remains somewhat enigmatic. This snail is tolerant of cold climate and is able to live farther north than in Transbaikalia.

According to archaeological and radiocarbon dating, the existence of extirpated mollusc species in the southeast of Transbaikalia does not correspond with the stages of the climatic time scale developed for the whole region (see Table 2). It possibly indicates the different palaeogeographic situation and the magnitude of climate change in the Pleistocene and Holocene in particular subregions of Transbaikalia.

During the periods of the Neopleistocene and Holocene climatic optima, five regionally extirpated mollusc species lived in the Upper Amur River Basin, in the territory of Transbaikalia, (Fig 12). Three of them (*Monodacna* cf. *colorata*, *M*. cf. *polymorpha*, and *Planorbis planorbis*) disappeared during the Neopleistocene, whereas *Lanceolaria grayii* and *Amuropaludina praerosa* were present in the region until the mid-Holocene. In our opinion, these species may be considered more or less reliable indicators of the climate change in the Transbaikalia region.

Changes in the species composition of the malacofauna of Transbaikalia associated with long- and short-term natural cycles of warming in the interglacial and cooling periods during the Neopleistocene and Holocene can be considered as a manifestation of climatogenic succession. During this succession, there was a decrease in the species diversity of molluscs at the regional level and a reduction in the peripheral areas of the ranges of thermophilic species at the global level.

## Supporting information

S1 Fig Subfossil specimens of the margaritiferid mussels. Shells of *M*. *dahurica* from excavation of an ancient site near the Darasun settlement (1620±60 years BP). A-C–reconstruction of shell length by overlapping some fossil shell fragments with valves of the recent *M*. *dahurica* from the Ingoda River.
(JPG)

S2 Fig Shells of *M*. *dahurica* from excavation of medieval Proezzhaya 1 fortress (1550±80 years BP). A –from the bank of defensive fortification, B –from the ancient dwelling 32 and reconstruction of shell length by superposition with the recent shell valves of *M*. *dahurica* from the Shilka River.
(JPG)

S3 Fig Shells of *M*. *dahurica* from excavation of the ancient settlement near the mouth of the Zheltuga River (1770±90 years BP). Scale bar 2 cm.
(JPG)

S4 Fig Fragments of shell valves and whole shells of *M*. *dahurica* from excavation of the Bol’shaya Kanga ancient settlement (2080±70 years BP), and reconstruction of fossil shell length by superposition with the recent shells of *M*. *dahurica* from the Argun River. (JPG)

S5 Fig Handmade goods from the mollusc shell. A –a button from nacre and blanks for beads and bracelet, B –arrowheads an d spearheads, C –adornments in the form of the pendant.
(JPG)
