# Supplementary figures and images for: The Pleistocene-Holocene aquatic molluscs as indicators of the past ecosystem changes in Transbaikalia (Eastern Siberia, Russia)
Source: PLoS One. 2020 Sep 18;15(9):e0235588. doi: 10.1371/journal.pone.0235588 (PMC7500642; doi:10.1371/journal.pone.0235588)

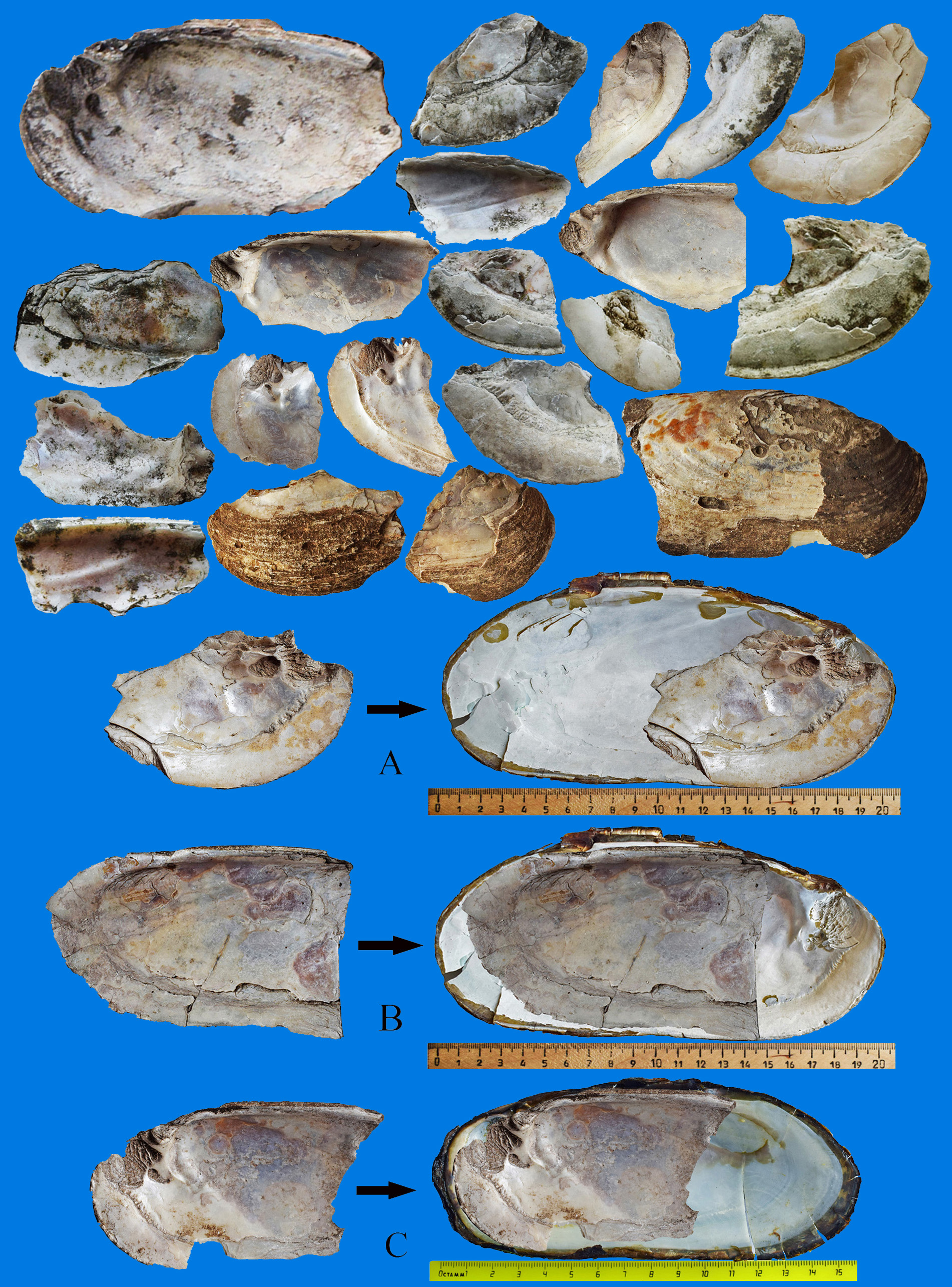

Supplement: S1 Fig — Shells of M. dahurica from excavation of an ancient site near the Darasun settlement (1620±60 years BP). A-C–reconstruction of shell length by overlapping some fossil shell fragments with valves of the recent M. dahurica from the Ingoda River. (JPG) [file pone.0235588.s001.jpg]

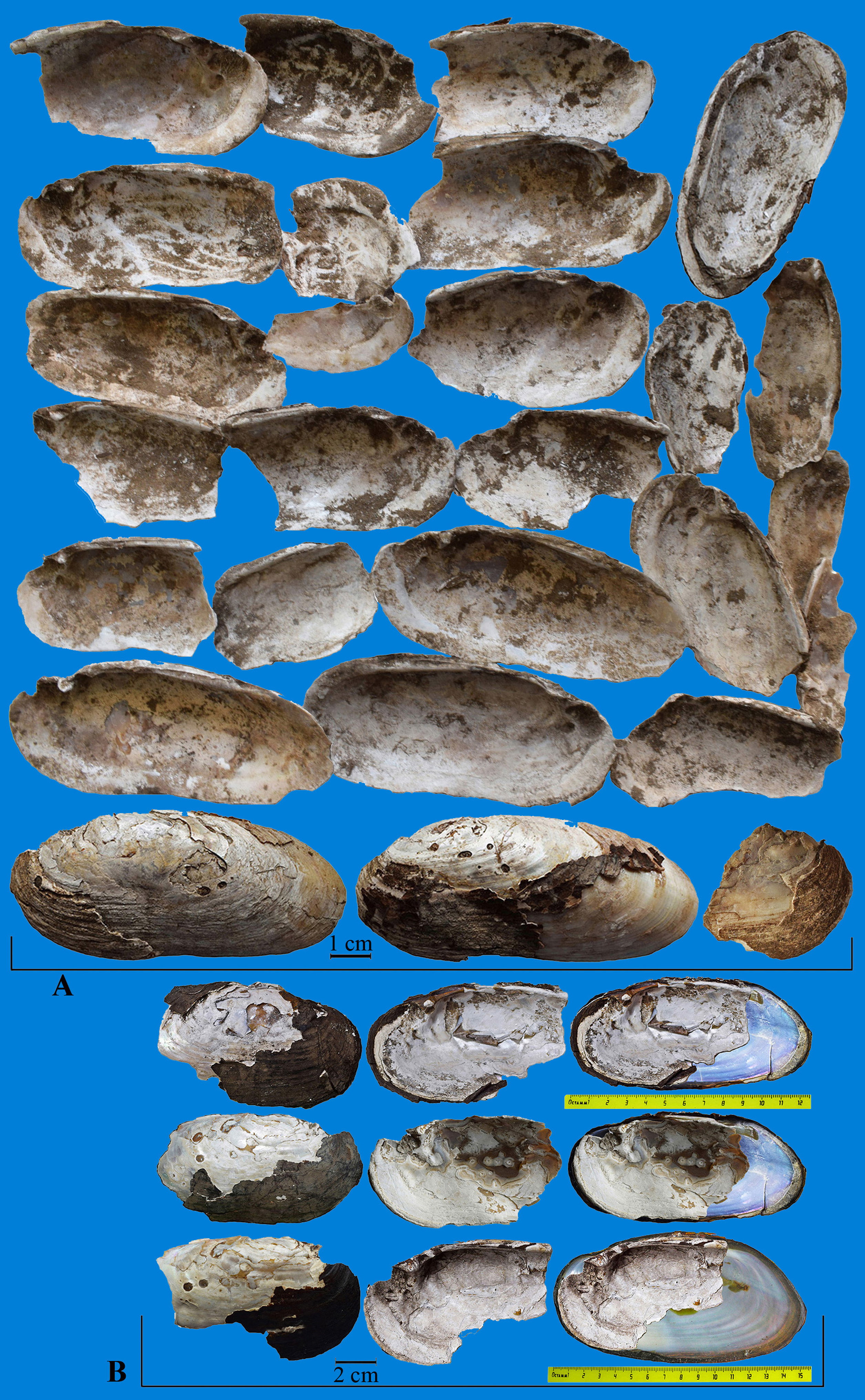

Supplement: S2 Fig — A –from the bank of defensive fortification, B –from the ancient dwelling 32 and reconstruction of shell length by superposition with the recent shell valves of M. dahurica from the Shilka River. (JPG) [file pone.0235588.s002.jpg]

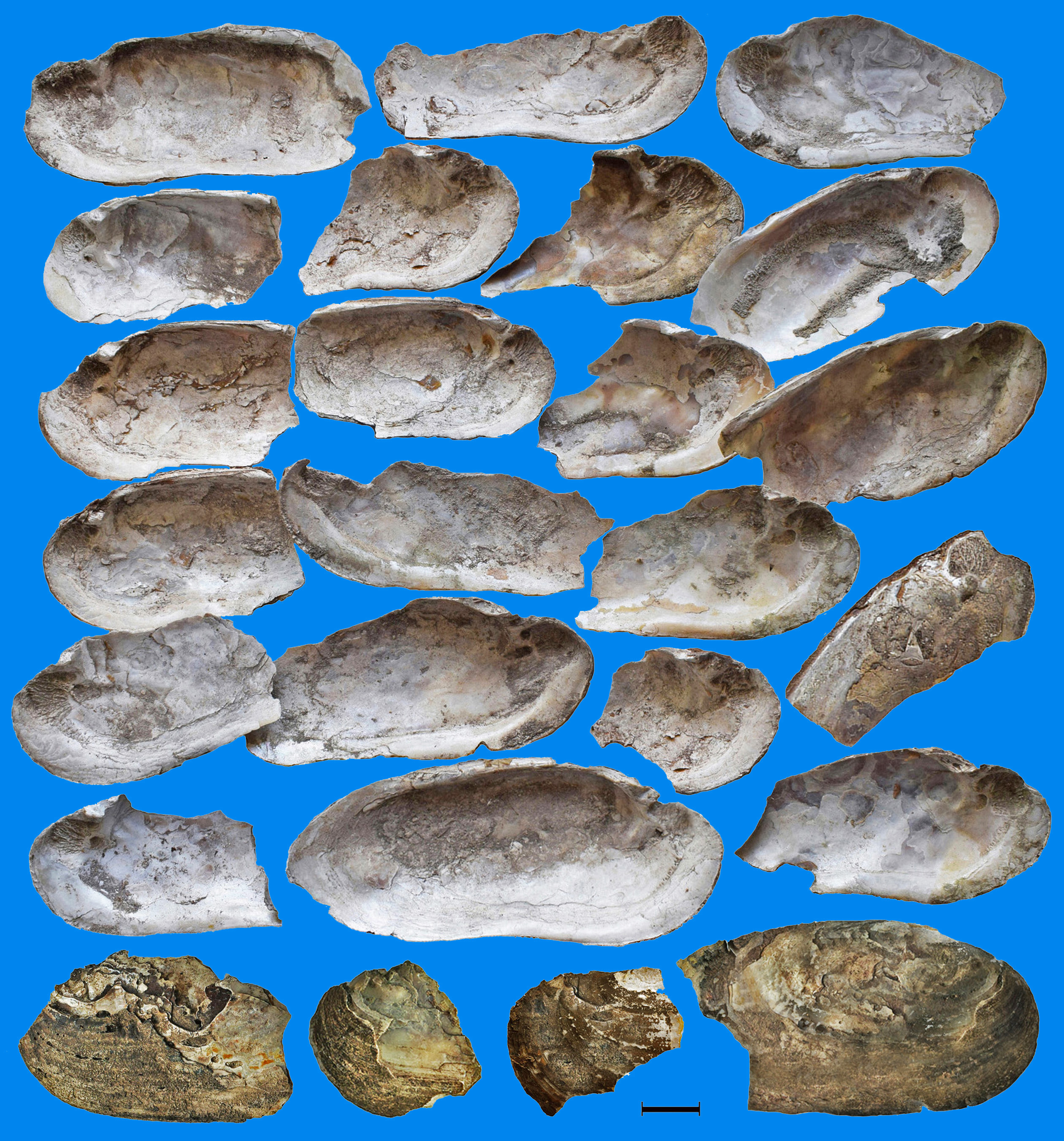

Supplement: S3 Fig — Scale bar 2 cm. (JPG) [file pone.0235588.s003.jpg]

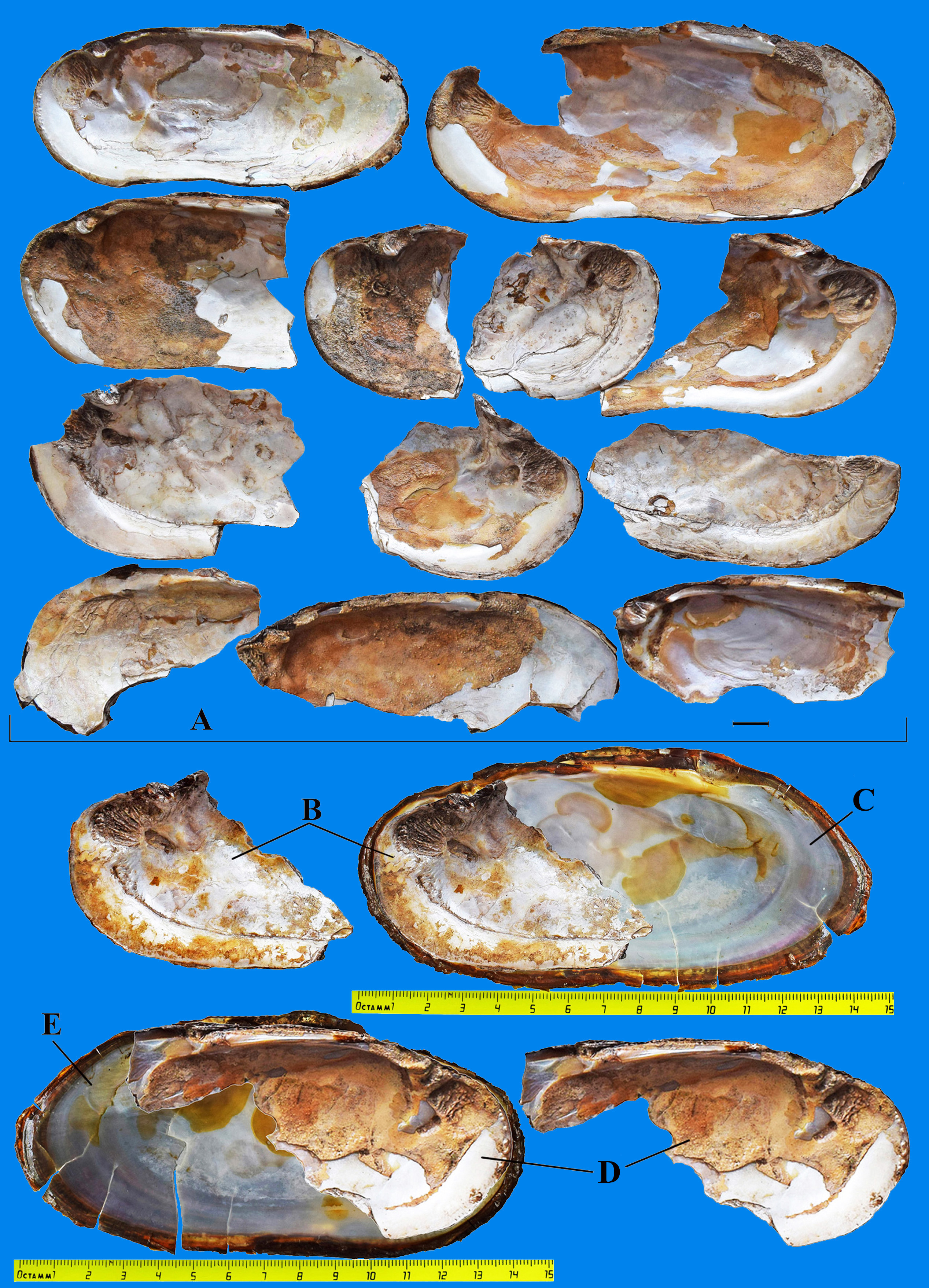

Supplement: S4 Fig — (JPG) [file pone.0235588.s004.jpg]

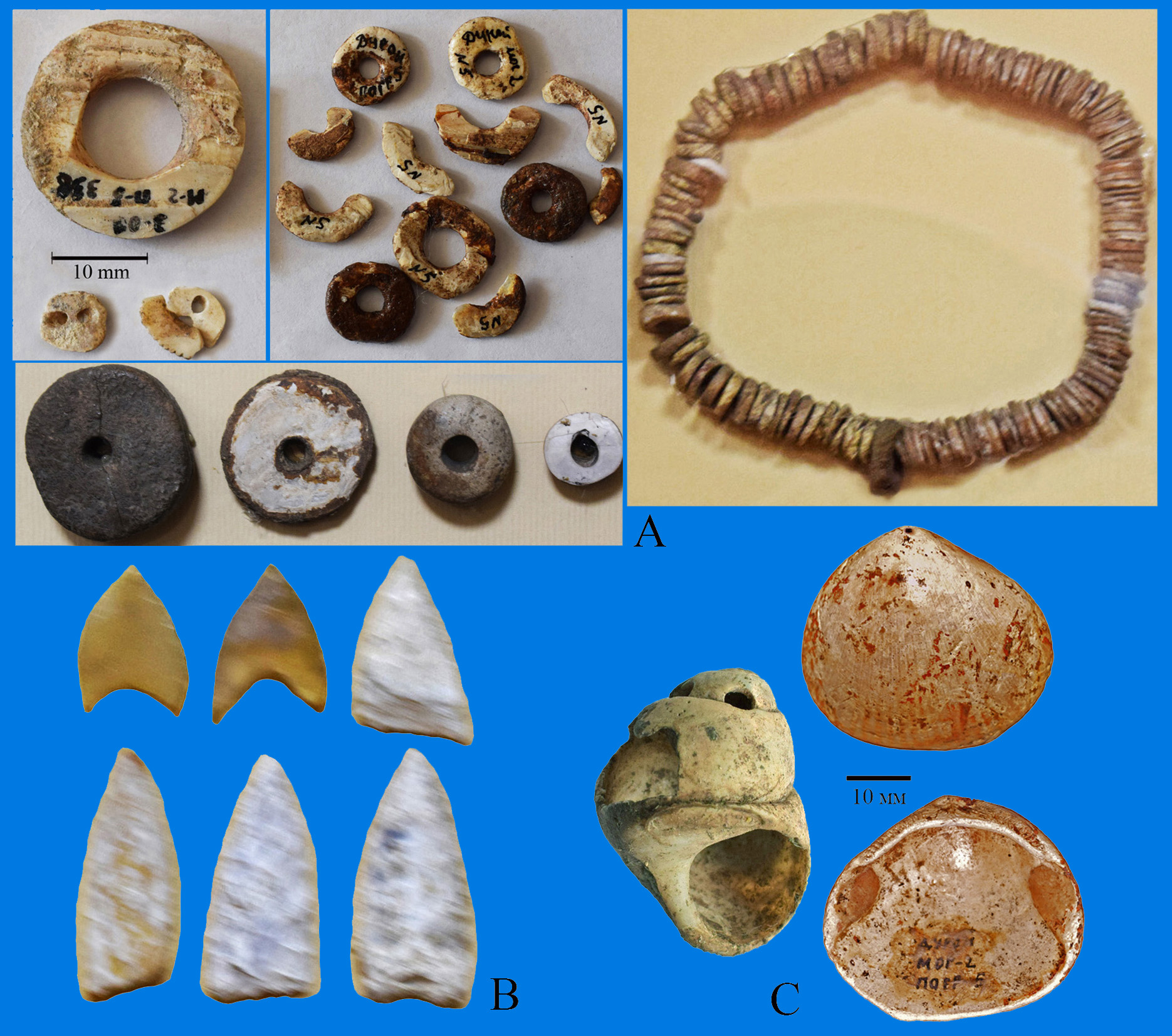

Supplement: S5 Fig — A –a button from nacre and blanks for beads and bracelet, B –arrowheads an d spearheads, C –adornments in the form of the pendant. (JPG) [file pone.0235588.s005.jpg]
